# Affective Organizational Commitment Links Job Satisfaction to Quiet Quitting in Healthcare Workers: The Amplifying Role of Career Engagement

**DOI:** 10.3390/healthcare14152366

**Published:** 2026-08-03

**Authors:** Sümeyra Gündem, Selma Söyük, Salim Yılmaz

**Affiliations:** 1 Department of Healthcare Management, Faculty of Health Sciences, Istanbul Arel University, Istanbul 34010, Türkiye; sumeyraefe@arel.edu.tr; 2 Department of Healthcare Management, Institute of Graduate Studies, Istanbul University-Cerrahpasa, Istanbul 34320, Türkiye; 3 Department of Health Management, Faculty of Health Sciences, Istanbul University-Cerrahpasa, Istanbul 34500, Türkiye; ssoyuk@iuc.edu.tr; 4 Department of Healthcare Management, Faculty of Health Sciences, Acibadem Mehmet Ali Aydinlar University, Istanbul 34752, Türkiye; 5 Department of Healthcare Management, Graduate School of Health Sciences, Acibadem Mehmet Ali Aydinlar University, Istanbul 34752, Türkiye

**Keywords:** quiet quitting, job satisfaction, affective organizational commitment, career engagement, healthcare workers

## Abstract

**Background/Objectives**: This study examined the relationships among job satisfaction, affective organizational commitment, career engagement, and quiet quitting in healthcare workers. Specifically, we tested a moderated mediation model in which affective organizational commitment mediated the relationship between job satisfaction and quiet quitting and career engagement moderated this indirect effect. **Methods**: In this cross-sectional study, data were collected online between April and May 2024 from 373 employees of public and private healthcare institutions in Istanbul, Türkiye. Participants completed validated measures of the four constructs. We tested mediation and moderation with SPSS 26.0 and the Hayes PROCESS macro (Model 14). **Results**: Job satisfaction correlated positively with affective organizational commitment and strongly and negatively with quiet quitting. Quiet quitting was higher among women (d = 0.42); nurses descriptively showed the lowest job satisfaction and the highest quiet quitting; however, no pairwise difference in quiet quitting was detected. The conditional indirect association between job satisfaction and quiet quitting through affective organizational commitment became stronger as career engagement increased. **Conclusions**: Job satisfaction and affective organizational commitment emerged as key correlates of lower quiet quitting. Career engagement amplified rather than buffered the role of affective organizational commitment: commitment was most strongly associated with lower quiet quitting among highly career-engaged employees, whereas low commitment in this group was accompanied by higher quiet quitting. Healthcare managers may consider strategies that strengthen employee commitment while supporting career development.

## 1. Introduction

Although quiet quitting has entered the literature only recently, the phenomenon itself is old. It manifests as exerting the minimum energy required to keep the job without resigning, incomplete performance of the job, and low productivity, and it returns to the organization as physical and psychological harm [1]. According to Gallup polls, in the first quarter of 2022 more than half of the working population in the US showed high levels of quiet quitting, the lowest employee engagement reported in recent years [2]. This trend intensified after COVID-19 because of burnout and health anxieties [3]. Many factors drive quiet quitting, including perceived low wages, feeling unappreciated, weak connection with the organizational purpose, and a toxic work environment. Because quiet quitting is regarded as a precursor of low productivity and active turnover, organizations need proactive strategies [4].

Yasin et al. [5] conceptualized quiet quitting as a dilemma in which employees meet institutional expectations at a minimum level while withdrawing psychologically. The concept erodes the commitment employees feel toward their institution [6], and in the health sector researchers have cautioned that its consequences may ultimately bear on care quality and patient safety [7], a possibility that underscores its importance even though such outcomes were not examined in the present study. Recent studies in healthcare settings confirm that quiet quitting is widespread among nurses and other health professionals, underscoring the need to understand its correlates [8,9,10].

Job satisfaction has been defined in many ways since its emergence, including the individual’s psychological attitude, emotional responses, and general thoughts toward the job. Most generally, it is an emotional and cognitive process that expresses the degree of satisfaction individuals derive from their working lives [11]. It is shaped not only by individual attitudes but also by the work environment, managerial attitude, the job itself, additional opportunities, and communication practices. Job satisfaction results from the positive and negative attitudes employees develop toward their jobs, and its primary determinant is the gap between what the institution offers and what employees expect from the job [12]. Studies have linked it to employee productivity, turnover intention, employee health, and affective organizational commitment [13].

Commitment both affects and is affected by job satisfaction. It is central to the development of institutional memory and the preservation of workforce stability [14], and organizations cannot ignore it if they wish to remain competitive and retain valuable employees. Meyer and Allen examined organizational commitment in three dimensions. In this study we focus on the affective component of organizational commitment, that is, employees’ emotional attachment to and identification with their organization, because this dimension is the most consistently related to job attitudes and withdrawal behaviors [15]. Affective commitment reflects identification with organizational goals and values and a strong bond with the organization. Employees with affective commitment stay by choice rather than obligation, which is why it is considered the gold standard. Continuance commitment arises when individuals remain in the institution because leaving would be costly, so they stay because they need to. Normative commitment is the loyalty employees feel toward the institution as a moral responsibility.

Working life increasingly requires proactive behavior, in which individuals anticipate job-related developments without waiting for an external stimulus, activate control mechanisms, initiate needed change, and adapt to it, and, in the career domain, develop the competencies required for advancement [16]. Career engagement extends proactivity to the career context and encompasses all proactive behaviors that develop an individual’s career, such as career planning, career exploration, networking, and self-development. What distinguishes it from related concepts is that it is behavior-based and observable rather than attitudinal [17]. Career engagement is thus a strategic building block that makes employees the agents of their own career journeys.

### 1.1. Theoretical Framework and Hypothesis Development

Conservation of resources (COR) theory [18,19] provides the integrative frame for the hypothesized model. COR holds that people strive to obtain, retain, and protect valued resources, that actual or threatened resource loss generates strain, and that individuals invest resources in order to gain further resources. Work attitudes can be read through this lens. Job satisfaction signals to employees that their investments of time and energy are being reciprocated, and it functions as an experienced resource gain at work. Quiet quitting, by contrast, can be understood as a resource-conservation strategy: when work is experienced as depleting rather than enriching, employees protect what remains by withholding discretionary effort while formally staying in the job. Satisfied employees should therefore have little need for such defensive withdrawal, whereas dissatisfied employees should resort to it more readily.

**H_1_.**  *Job satisfaction is negatively related to quiet quitting.*

COR also explains why this relationship should operate, in part, through affective organizational commitment. Sustained resource gains at work foster what the theory calls resource caravans, accumulations of interconnected resources that include the emotional bond between employee and organization. Job satisfaction thus builds affective attachment, and this attachment is itself a motivational resource: emotionally attached employees experience the organization’s goals as their own and continue investing effort in them, which leaves little room for the withdrawal that defines quiet quitting. Prior studies have documented the individual links in this chain [13,20], but the mediating pathway has rarely been tested as a whole in healthcare samples.

**H_2_.**  *Affective organizational commitment mediates the negative relationship between job satisfaction and quiet quitting.*

The central and least understood question, however, is conditional: for whom does low attachment translate into actual withdrawal? We propose that career engagement acts as a boundary condition. In COR terms, career-engaged employees hold a strong personal resource, namely agency over where their energies go, and they allocate that resource deliberately [21]. When such employees are also emotionally attached to their organization, their proactive energies are more likely to be directed toward the organization, thereby reducing the likelihood of withdrawal-related behavior [22]. When attachment is low, the same agency may facilitate a redirection of investments toward potentially transferable career resources through networking, exploration, and self-development [23], and withholding organizational effort may become one possible resource-protection response. Employees low in career engagement lack this allocation flexibility, so their effort depends less on how attached they feel. Career engagement should therefore amplify, rather than buffer, the negative relationship between affective organizational commitment and quiet quitting: it is a double-edged resource whose direction depends on where the employee’s attachment lies [24].

**H_3_.**  *Career engagement moderates the negative relationship between affective organizational commitment and quiet quitting, such that the relationship becomes stronger as career engagement increases.*

Because the commitment–quiet quitting link is conditional on career engagement, the indirect pathway from job satisfaction must be conditional as well [25,26,27].

**H_4_.**  *Career engagement moderates the indirect effect of job satisfaction on quiet quitting through affective organizational commitment, such that the indirect effect becomes stronger as career engagement increases.*

### 1.2. The Present Study

Against this background, the present study tested hypotheses H_1_–H_4_ in a sample of healthcare workers in Türkiye, using a moderated mediation design, an approach increasingly applied in health-related research, in which affective organizational commitment carries the effect of job satisfaction on quiet quitting and career engagement conditions the strength of that pathway.

In addition to hypotheses H_1_–H_4_, the study addressed two descriptive research questions: (i) What are the levels of job satisfaction, affective organizational commitment, career engagement, and quiet quitting among healthcare workers? (ii) Do these variables differ by demographic characteristics such as gender, education level, income, profession, and professional experience? The second question was exploratory, as no directional hypotheses were formulated for demographic differences.

## 2. Materials and Methods

### 2.1. Study Design

We conducted a descriptive, cross-sectional, correlational study and reported it in accordance with the STROBE (Strengthening the Reporting of Observational Studies in Epidemiology) guidelines for cross-sectional studies; the completed checklist is provided in Supplementary Table S1.

**Figure 1:**
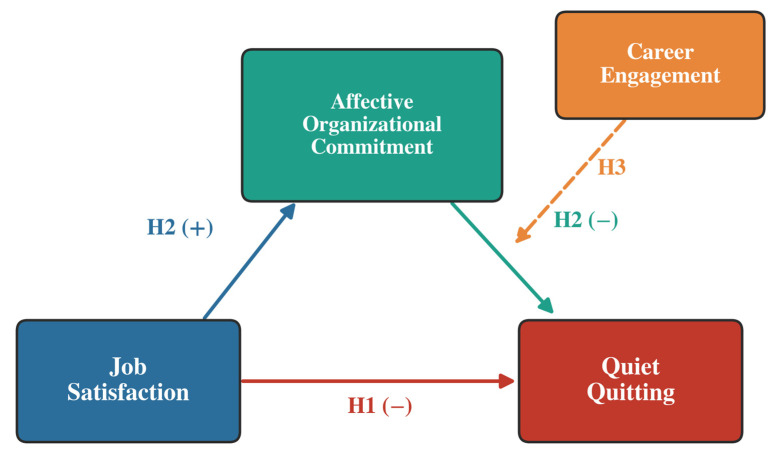
Hypothesized conceptual model. *Job satisfaction is expected to relate negatively to quiet quitting directly (H_1_) and indirectly through affective organizational commitment (H_2_). The dashed arrow denotes the hypothesized moderation: career engagement is expected to strengthen the negative relationship between affective organizational commitment and quiet quitting (H_3_), implying that the indirect effect of job satisfaction on quiet quitting is conditional on career engagement (H_4_).*

We specified the hypothesized moderated mediation model (Figure 1) according to Hayes [28] Model 14: job satisfaction served as the independent variable, quiet quitting as the dependent variable, affective organizational commitment as the mediator, and career engagement as the moderator.

### 2.2. Setting and Participants

The target population consisted of healthcare workers employed in public and private healthcare institutions in Istanbul, Türkiye’s largest metropolitan area. For national context, according to the 2023 Health Statistics Yearbook published by the Ministry of Health [29], Türkiye had 1,413,921 healthcare workers. Participants were recruited through convenience and snowball sampling. The survey link was shared through the authors’ workplace and personal professional networks, by telephone and through WhatsApp groups, and recipients were encouraged to forward it to other eligible healthcare workers. The invitation stated that only healthcare workers currently employed in Istanbul were eligible, and prospective participants from other provinces were not enrolled. Because the link circulated through personal networks, the number of individuals who received it could not be tracked, so a response rate cannot be calculated. Inclusion criteria were being 18 years of age or older and actively working in a public or private healthcare institution in Istanbul at the time of data collection. Although employees of both public and private institutions were represented, institution type was not recorded in the questionnaire and therefore could not be analyzed. Of the 383 healthcare workers who submitted the questionnaire, 10 were excluded because their forms contained unanswered items, leaving a final sample of 373 with complete data on all study variables.

Consistent with the conditional process analysis framework of Hayes [28], we calculated the required sample size a priori with G*Power 3.1.9.4 for linear multiple regression (fixed model, R^2^ deviation from zero) with four predictors, assuming a small-to-medium effect size (f^2^ = 0.05), α = 0.05, and power = 0.80. This calculation indicated a minimum of 244 participants [30]. We used a regression-based calculation because PROCESS Model 14 is estimated as a system of ordinary least squares equations and the outcome equation, which contains the focal interaction term, has four predictors; no closed-form power calculation exists for conditional indirect effects, so the most directly testable component of the model served as the planning target. A sensitivity analysis showed that the final sample of 373 provided 80% power to detect effects as small as f^2^ = 0.032, below the observed effect size of the interaction term (f^2^ = 0.08). We do not report post hoc power for the observed effects, because observed power is a direct function of the *p* value and adds no information beyond it; inference instead relies on the percentile bootstrap confidence intervals reported below [28].

### 2.3. Data Collection

We collected data online between April and May 2024 using Google Forms, distributed as described in Section 2.2. The first page presented the study information and the informed consent form, and the questionnaire opened only after consent was given; participation was voluntary and anonymous, and no identifying information was collected. The platform did not technically prevent multiple submissions; duplicate participation, although unlikely given the personal distribution channels, cannot be fully ruled out.

### 2.4. Measures

The questionnaire consisted of a demographic information form and four validated scales.

#### 2.4.1. Job Satisfaction Scale

The scale was originally developed by Brayfield and Rothe [31], and its five-item short form was constructed by Judge et al. [32]. The Turkish adaptation was carried out by Keser and Öngen Bilir [33]. The scale consists of five items in a single dimension, rated on a five-point Likert scale, and items 3 and 5 are reverse scored. Item scores are summed to a total ranging from 5 to 25; higher scores indicate greater job satisfaction.

#### 2.4.2. Affective Organizational Commitment Scale

The scale is based on the Affective Commitment Scale developed by Meyer et al. [34], and its Turkish form was prepared by Güney et al. [35]. The scale contains six items, of which items 3, 4, and 5 are reverse scored. In the present study, the items were rated on a five-point Likert scale. Total scores range from 6 to 30; higher scores indicate stronger affective commitment.

#### 2.4.3. Career Engagement Scale

The scale was developed by Hirschi et al. [36], and the Turkish adaptation was conducted by Korkmaz et al. [37]. The scale comprises nine items in a single dimension, rated from 1 (almost never) to 5 (very often). Total scores range from 9 to 45; higher scores indicate more frequent proactive career behaviors.

#### 2.4.4. Quiet Quitting Scale

The scale was developed by Avcı [38]. It consists of eight items grouped under two subdimensions, quiet quitting towards work (three items) and quiet quitting towards life (five items). In the original scale [38], the work-related items reflect negative appraisals of one’s standing and prospects at the current workplace (e.g., “I feel undervalued at the company where I work”), whereas the life-related items reflect the prioritization of personal life, health, and work–life boundaries over work demands (e.g., “My health and well-being come before my career”); full item-level statistics are provided in Supplementary Tables S5 and S6. There are no reverse-scored items, and responses are given on a five-point Likert scale. Total scores range from 8 to 40; higher scores indicate greater quiet quitting. In the present sample, the two subdimensions were substantially correlated (ρ = 0.58) and showed the same pattern of associations with the other study variables, and the life-related items themselves correlated substantially with the work attitudes examined here (mean |ρ| = 0.45 with job satisfaction and 0.50 with affective organizational commitment; Supplementary Table S6); because the original scale is scored as a total and the study hypotheses concern overall quiet quitting, the total score was used in the main analyses, and subdimension-level results are additionally reported.

All four instruments are measures whose linguistic validity and psychometric properties had been established in Turkish samples through prior adaptation or development studies, in line with methodological recommendations favoring the use of systematically adapted and validated instruments over ad hoc translations [39]. In this study, the Cronbach’s alpha coefficients were found to be 0.91 for job satisfaction, 0.90 for affective organizational commitment, 0.94 for career engagement, and 0.87 for quiet quitting (0.64 for the work-related and 0.88 for the life-related subdimension). McDonald’s omega was 0.69 for the work-related and 0.88 for the life-related subdimension. The modest internal consistency of the three-item work-related subdimension reflected its first item: the mean inter-item correlation was 0.37, the corrected item–total correlations were 0.25, 0.57, and 0.56, and alpha rose to 0.78 when the first item was removed (Supplementary Table S5).

### 2.5. Statistical Analysis

We analyzed the data using IBM SPSS 26.0, the Hayes PROCESS macro (Model 14), and R (version 4.6) with the lavaan [40] and semTools packages. We examined normality with the Shapiro–Wilk test together with skewness, kurtosis, and Q–Q plots (Supplementary Figure S3); because the score distributions deviated from normality, we used Spearman correlations. Demographic comparisons were exploratory, as no hypotheses were formulated for them. We tested group differences with the independent-samples t test and one-way analysis of variance (ANOVA), reporting Cohen’s d and omega-squared as effect sizes, and we applied the Games–Howell post hoc test with 95% confidence intervals when variance homogeneity was not achieved. Because several subgroups were small and some distributions were skewed, we repeated all comparisons with Mann–Whitney U, Welch ANOVA, and Kruskal–Wallis tests as sensitivity analyses, and we report Holm-adjusted *p* values across the full set of 28 omnibus tests. We evaluated the mediating effect of affective organizational commitment and the moderating effect of career engagement on the indirect effect of job satisfaction on quiet quitting with PROCESS Model 14. In the analyses, we used 5000 bootstrap samples and computed 95% percentile bootstrap confidence intervals. We probed conditional effects at the 16th, 50th, and 84th percentiles of the moderator. Prior to hypothesis testing, we evaluated the measurement model with confirmatory factor analysis (CFA). Because item distributions deviated from normality, we used maximum likelihood estimation with robust standard errors and a Satorra–Bentler scaled test statistic (MLR) and report robust fit indices, interpreting CFI and TLI ≥ 0.90 together with RMSEA and SRMR ≤ 0.08 as indicating acceptable fit [41]. The hypothesized measurement model was compared with more constrained alternative models using scaled chi-square difference tests and information criteria. The tenability of a total quiet-quitting score was additionally evaluated with a higher-order model, in which the two subdimensions loaded on a general quiet-quitting factor, and with bifactor model indices (hierarchical omega, ωH, and explained common variance, ECV). Convergent and discriminant validity were assessed with standardized loadings, composite reliability (CR), average variance extracted (AVE) [42], and the heterotrait–monotrait ratio of correlations (HTMT < 0.85) [43], for which percentile bootstrap 95% confidence intervals (5000 samples) were computed; estimation treating the items as ordinal (WLSMV) served as a sensitivity check. To address common method bias, we combined procedural remedies (anonymous and voluntary participation, previously validated instruments) with statistical checks: Harman’s single-factor test (a single factor accounted for 44.5% of the variance), the fit of a single-factor CFA model, and an unmeasured latent method factor model [44]. Statistical significance was set at *p* < 0.05 (two-tailed).

Before forming the product term, the mediator and the moderator were mean-centered, which leaves the interaction coefficient and model fit unchanged but renders the lower-order coefficients interpretable as conditional effects at average levels and removes nonessential multicollinearity. We examined multicollinearity with variance inflation factors, residual normality, heteroscedasticity with the Breusch–Pagan and non-constant variance score tests, and influential observations with Cook’s distance, and we re-estimated the models with HC3 heteroscedasticity-consistent standard errors. The region of significance for the conditional effect of commitment was probed with the Johnson–Neyman technique [28]. Robustness was further assessed with five sensitivity analyses: a covariate-adjusted model with gender, profession (nurse versus other), education, income, and professional experience entered in both equations, models using each quiet-quitting subdimension as the outcome, re-estimation excluding influential cases, re-estimation excluding the weakly loading quiet-quitting item, and re-estimation using factor scores from the five-factor measurement model, with the general quiet-quitting factor from the bifactor model as the outcome.

## 3. Results

The mean age of the participants was 33.81 ± 8.82 years (range 19–61) (Table 1).

Confirmatory factor analysis supported the hypothesized measurement structure (Table 2). The five-factor model, in which quiet quitting was represented by its two correlated subdimensions, fit the data acceptably (χ^2^(340) = 908.78, CFI = 0.913, TLI = 0.904, RMSEA = 0.072, 90% CI [0.067, 0.078], SRMR = 0.063) and fit significantly better than a four-factor model treating quiet quitting as unidimensional (Δχ^2^(4) = 96.75, *p* < 0.001). All constructs were empirically distinguishable: merging affective organizational commitment with quiet quitting (Δχ^2^(3) = 90.21, *p* < 0.001), merging job satisfaction with affective organizational commitment (Δχ^2^(3) = 110.91, *p* < 0.001), and a single-factor model (Δχ^2^(6) = 651.86, *p* < 0.001; CFI = 0.624, RMSEA = 0.148) all worsened fit significantly. Consistent with the use of a total score, a higher-order model in which the two quiet-quitting subdimensions loaded on a general quiet-quitting factor fit the data as well as the correlated five-factor model (Δχ^2^(2) = 1.33, *p* = 0.514; ΔCFI < 0.001; standardized higher-order loadings 0.99 and 0.80), and a bifactor model of the quiet-quitting items indicated a dominant general factor (ωH = 0.76, ECV = 0.72) with little reliable specific variance beyond it (ωHS = 0.15 for the work-related and 0.24 for the life-related subdimension; Supplementary Table S5). Standardized loadings were significant (all *p* < 0.001) and ranged from 0.62 to 0.91, except one work-related quiet-quitting item (λ = 0.33; Supplementary Table S3). This item correlated weakly with all other quiet-quitting items (r = 0.21–0.27), and its largest modification index pointed to a negative cross-loading on career engagement rather than to the other quiet-quitting factor, indicating diffuse rather than subdimension-specific weakness. When the item was removed, model fit was essentially unchanged (five-factor CFI = 0.917, RMSEA = 0.073) while the psychometric indices of the affected factors improved (quiet-quitting AVE = 0.54; work-related subdimension CR = 0.78, AVE = 0.64; Spearman–Brown reliability of the remaining two items = 0.78; Supplementary Table S5). The item was nevertheless retained in the primary analyses to preserve the validated scoring of the original instrument and comparability with prior studies, and all substantive conclusions were confirmed with the item excluded (see robustness checks below). Composite reliability was high for all scales (CR ≥ 0.88), and AVE exceeded 0.60 for job satisfaction, affective organizational commitment, and career engagement; for quiet quitting, AVE (0.48) fell marginally below 0.50, reflecting the weaker item, and the work-related subdimension containing this item showed the same pattern (AVE = 0.46). Given the strong latent correlations (e.g., −0.81 between affective organizational commitment and quiet quitting), the Fornell–Larcker criterion was not satisfied for the most highly correlated construct pairs. At the level of the four composite constructs used in the structural analyses, all HTMT point estimates were below the 0.85 threshold (largest = 0.82), although the upper bootstrap confidence limits for the two strongest pairs—job satisfaction–commitment and commitment–quiet quitting—slightly exceeded 0.85 (both 0.87) while remaining below 0.90 (full matrix in Supplementary Table S4), the confidence intervals of the latent correlations excluded unity (e.g., commitment–quiet quitting −0.81, 95% CI [−0.87, −0.76]), and merging the constructs worsened fit significantly, including a targeted model combining affective organizational commitment with work-related quiet quitting (Δχ^2^(4) = 37.95, *p* < 0.001). At the subdimension level, however, the HTMT value for affective organizational commitment and work-related quiet quitting reached 0.93 (95% CI [0.86, 1.00]), exceeding both conventional thresholds, and in an exploratory bifactor model of the commitment and quiet-quitting items a common factor accounted for 76% of the shared item variance. The constructs can therefore be treated as related but distinct at the level analyzed here, while the distinctness of commitment from work-related quiet quitting in particular is relative rather than absolute; the implications are considered in the Discussion. A sensitivity analysis treating the items as ordinal (WLSMV) produced strong CFI, TLI, and SRMR values (CFI = 0.956, TLI = 0.951, SRMR = 0.058), whereas RMSEA (0.086, 90% CI [0.081, 0.091]) was slightly above the prespecified 0.08 threshold, thereby providing partial rather than uniformly strong support for the factor structure. Consistent with limited common method variance, the single-factor model fit poorly, and an orthogonal, equal-loading latent method factor accounted for less than 1% of the shared variance and did not improve model fit (Δχ^2^(1) = 0.45, *p* = 0.50, ΔCFI < 0.001). Even so, all constructs were self-reported within a single survey, so common method variance cannot be ruled out entirely, and the strength of the observed associations should be interpreted with this in mind.

The mean scores were 16.14 ± 5.20 for job satisfaction (range 5–25), 18.69 ± 6.28 for affective organizational commitment (6–30), 29.28 ± 8.81 for career engagement (9–45), and 27.37 ± 7.37 for quiet quitting (8–40). Spearman correlation analysis revealed significant associations among all study variables (n = 373, all *p* < 0.001). Job satisfaction was positively correlated with affective organizational commitment (ρ = 0.722) and career engagement (ρ = 0.497) and negatively correlated with quiet quitting (ρ = −0.703). Affective organizational commitment was positively correlated with career engagement (ρ = 0.442) and negatively correlated with quiet quitting (ρ = −0.756). Career engagement was negatively correlated with quiet quitting (ρ = −0.454) (Table 3).

Demographic comparisons were exploratory and are summarized in Table 4 together with effect sizes; pairwise Games–Howell contrasts with 95% confidence intervals appear in Supplementary Table S2. Job satisfaction differed significantly by gender, with men scoring higher (t(371) = −2.69, *p* = 0.007, d = 0.31). Men also reported higher affective organizational commitment (t(371) = −2.25, *p* = 0.025, d = 0.26), whereas quiet quitting was higher among women (Welch’s t(160) = 3.40, *p* = 0.001, d = 0.42); all three differences were confirmed by Mann–Whitney tests. Career engagement did not differ by gender (*p* = 0.424). Marital status was not associated with job satisfaction, commitment, career engagement, or quiet quitting (all *p* > 0.05). Education level did not differentiate job satisfaction or commitment. Although the ANOVA indicated an overall difference in quiet quitting by education (F(4, 368) = 3.60, *p* = 0.007), no pairwise comparison (Games–Howell) reached significance, and the omnibus difference was not corroborated by the Welch ANOVA (*p* = 0.065) or the Kruskal–Wallis test (*p* = 0.184).

Profession was associated primarily with job satisfaction (ω^2^ = 0.055) and affective organizational commitment (ω^2^ = 0.042), and these differences were consistent across Welch and Kruskal–Wallis tests. Descriptively, nurses had the lowest job satisfaction and the highest quiet quitting; however, the only significant pairwise difference was between nurses and the heterogeneous “other” group (civil servants, managers, etc.), whose job satisfaction and affective organizational commitment were higher (Games–Howell, *p* < 0.05). For quiet quitting, the omnibus ANOVA was significant but not robust (Welch *p* = 0.086) and no pairwise comparison reached significance, so profession-based differences in quiet quitting should not be overinterpreted. Regarding income, job satisfaction and affective organizational commitment were higher in the 12,001–24,000 TL group than in the 24,001–36,000 TL group, and quiet quitting was higher in the 24,001–36,000 TL group than in the two highest income groups. Total professional experience also produced significant differences. Employees with 1–5 years of experience had the lowest job satisfaction (M = 14.92) and affective organizational commitment (M = 16.61). Their job satisfaction was significantly lower than in the 16–20 years and 21-years-and-above groups, and their affective organizational commitment was significantly lower than in the less-than-1-year, 16–20 years, and 21-years-and-above groups (Games–Howell, *p* < 0.05). For quiet quitting, the omnibus ANOVA was significant, but no pairwise comparison reached significance. Descriptively, quiet quitting was highest in the 1–5 years group (M = 29.12) and lowest among employees with less than 1 year of experience (M = 24.71). No variable differed by tenure in the current institution (Table 4).

As summarized in Table 4, significant group differences were concentrated in gender, profession, income, and total professional experience, whereas marital status and institutional tenure did not differentiate any of the study variables. Descriptively, the least favorable profile, combining lower job satisfaction and affective organizational commitment with higher levels of quiet quitting, was observed among nurses, employees with 1–5 years of professional experience, and the middle-income group. When Holm correction was applied across the 28 exploratory omnibus tests, five associations remained significant: affective organizational commitment by professional experience and by profession, job satisfaction by profession, and quiet quitting by income and by gender; the remaining group differences should therefore be interpreted with particular caution.

We tested the moderated mediation model with Hayes PROCESS Model 14 [26,27,28], with the mediator and moderator mean-centered, using 5000 bootstrap samples and 95% percentile bootstrap confidence intervals. Indirect effects were considered significant when the confidence interval excluded zero (Table 5).

**Figure 2:**
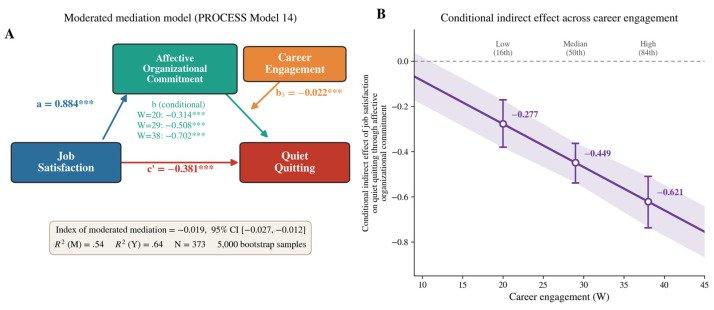
Moderated mediation model and conditional indirect effects. Panel (**A**) shows the statistical model (PROCESS Model 14) with unstandardized coefficients. Panel (**B**) shows the conditional indirect effect of job satisfaction on quiet quitting through affective organizational commitment across levels of career engagement, with 95% percentile bootstrap confidence intervals (5000 samples). N = 373. *** *p* < 0.001.

In the second stage of the analysis, the conditional indirect effects of job satisfaction on quiet quitting through affective organizational commitment were estimated at low (16th percentile), medium (50th percentile), and high (84th percentile) levels of career engagement (Figure 2).

Job satisfaction was strongly associated with affective organizational commitment (B = 0.884; *p* < 0.001). In the quiet quitting equation, job satisfaction had a significant direct negative effect (B = −0.381, *p* < 0.001). The affective organizational commitment × career engagement interaction was also negative and significant (B = −0.0216, *p* < 0.001). Accordingly, the effect of affective organizational commitment on quiet quitting depends on career engagement: the conditional effect of commitment was significant and negative at the 16th, 50th, and 84th percentiles of career engagement (W = 20, 29, and 38), with B = −0.314, −0.508, and −0.702, respectively (all *p* < 0.001). With mean-centered predictors, the lower-order coefficients are directly interpretable. At average career engagement, affective organizational commitment was negatively related to quiet quitting (B = −0.514, SE = 0.055, *p* < 0.001), and at average commitment, career engagement showed a smaller negative conditional effect (B = −0.146, SE = 0.030, *p* < 0.001). After centering, all variance inflation factors were at or below 2.3. The Johnson–Neyman analysis indicated that the conditional effect of commitment on quiet quitting was significantly negative for career engagement values of approximately 13.3 and above, a region covering 96.8% of the sample; the corresponding simple slopes are shown in Supplementary Figure S2. As illustrated in Figure 2 (Panel B), the indirect effect of job satisfaction on quiet quitting through affective organizational commitment also strengthened as career engagement increased (W20: −0.277; W29: −0.449; W38: −0.621). The index of moderated mediation was −0.019, 95% CI [−0.027, −0.012], which confirmed the moderated mediation. Across the entire observed range of career engagement, the conditional indirect effect remained below zero, indicating that the inverse indirect pathway was present at all levels of career engagement and grew progressively stronger at higher levels.

Because the conditional indirect effect strengthens with career engagement while the direct effect of job satisfaction remains constant, the relative contribution of the indirect pathway necessarily grows at higher levels of career engagement. We do not quantify this pattern as a mediated proportion, because ratio-based measures are unstable in bootstrap samples and the definition of the total effect is not unique in a moderated mediation model; the conditional indirect effects and the index of moderated mediation reported above constitute the primary evidence.

A series of robustness checks supported these conclusions. Regression diagnostics indicated mild deviations from residual normality (skewness = −0.16, kurtosis = 1.10) and significant heteroscedasticity (Breusch–Pagan test, *p* < 0.001); all coefficients remained significant with HC3 heteroscedasticity-consistent standard errors, and excluding 25 influential observations (Cook’s distance > 4/n) left the estimates essentially unchanged (interaction B = −0.023, *p* < 0.001; residual diagnostics appear in Supplementary Figure S1). The moderated mediation also held when gender, profession (nurse versus other), education, income, and professional experience were included as covariates in both equations (index of moderated mediation = −0.0155, 95% CI [−0.0226, −0.0086]), when each quiet-quitting subdimension served as the outcome (index = −0.0029, 95% CI [−0.0055, −0.0005] for the work-related and −0.0163, 95% CI [−0.0224, −0.0101] for the life-related subdimension, with a weaker interaction for the work-related dimension, B = −0.0033, *p* = 0.0496), when the weakly loading item was excluded from the quiet-quitting total (index = −0.0183, 95% CI [−0.0254, −0.0114]), and when the model was re-estimated with factor scores from the five-factor measurement model, using the general quiet-quitting factor from the bifactor model as the outcome (interaction B = −0.137, *p* < 0.001; index of moderated mediation = −0.116, 95% CI [−0.155, −0.077]).

## 4. Discussion

In this study, we examined the relationships among job satisfaction, affective organizational commitment, career engagement, and quiet quitting in healthcare workers. The findings show significant relationships among these variables in both direction and strength.

The bivariate pattern was consistent with prior work. Job satisfaction was positively associated with affective organizational commitment and with career engagement, in line with meta-analytic evidence on affective commitment [45] and with the reported association between job satisfaction and employee motivation and long-term career goals [46,47]. Job satisfaction and affective organizational commitment were both strongly and negatively associated with quiet quitting, and career engagement moderately so, echoing studies in which employees reported more quiet quitting when satisfaction was low [48,49] or when organizational support was lacking and burnout was high [9], less psychological distancing when commitment, trust, and belonging were high [50], and lower quiet quitting among career-oriented employees [51,52].

Regarding professional experience, employees with less than 1 year of experience reported relatively high job satisfaction and affective organizational commitment together with the lowest level of quiet quitting. A trough followed in the 1–5 years group, which showed the lowest job satisfaction and affective organizational commitment and, descriptively, the highest level of quiet quitting. Employees with 21 or more years of experience again showed higher job satisfaction and affective organizational commitment and lower quiet quitting. This cross-sectional trajectory echoes the honeymoon–hangover pattern described in longitudinal research [53]; however, because our data compare different individuals rather than following the same employees over time, the pattern may equally reflect cohort differences or the selective attrition of dissatisfied employees, and it should not be read as evidence of within-person change. Although Galanis et al. [54] associated greater clinical experience with lower quiet quitting, Moisoglou et al. [10] found no adjusted association between experience and work engagement or quiet quitting. Kooij et al. [55] further suggest that age-related motivational changes are multidimensional rather than uniformly positive. Consistent with this mixed evidence, career engagement did not differ by professional experience in the present study.

The moderation analysis (Model 14) showed that career engagement moderates the association between affective organizational commitment and quiet quitting and, in turn, the conditional indirect association of job satisfaction with quiet quitting through affective organizational commitment. The conditional indirect association between job satisfaction and quiet quitting through affective organizational commitment thus became stronger at higher levels of career engagement. This finding is consistent with recent evidence conceptualizing quiet quitting as a deliberate contraction of role boundaries rather than passive neglect. In a scale validation study conducted with primary healthcare workers, quiet quitting behavior was negatively correlated with the proactiveness dimension of organizational citizenship behavior and positively correlated with turnover intention [56], which is consistent with the negative bivariate association between career engagement and quiet quitting observed here.

Prior healthcare studies have linked quiet quitting to lower satisfaction and weaker attachment to the organization [10], but these associations have generally been examined separately; the present model ties them together by showing that the satisfaction–quiet quitting association operates partly through affective organizational commitment and is conditional on career engagement. The amplification pattern deserves theoretical attention, because career engagement is often assumed to be uniformly beneficial. Interpreted through the conservation of resources lens introduced in Section 1.1, career engagement functioned as a double-edged resource. Proactive career behaviors give employees agency over where their energies are invested, and affective organizational commitment appears to determine the direction of that investment. Among highly career-engaged employees, commitment was more strongly associated with lower quiet quitting; when commitment was low, the same employees showed the highest quiet quitting. One possible interpretation is a redirection of effort toward portable career resources, a reading that would align with the employability management paradox, in which highly employable, career-invested employees remain organizational assets primarily when they feel attached [57], and with boundaryless and protean career perspectives, in which self-directed employees calibrate organizational effort against their own career agendas [58]. Because external employability, job-search intentions, career orientations, and actual resource allocation were not measured, however, these accounts remain plausible interpretations rather than demonstrated mechanisms, and highly career-engaged employees may equally have been pursuing advancement within their organizations. In practical terms, career engagement amplified the importance of affective organizational commitment rather than substituting for it.

Overall, job satisfaction, affective organizational commitment, and career engagement were associated with lower quiet quitting. Among the experience-related patterns specifically, only the difference in affective organizational commitment survived multiplicity adjustment (the full set of Holm-robust associations is listed in the Results); the other experience-related differences, including the descriptive trough among employees with 1–5 years of experience, should be regarded as exploratory signals for future research rather than a basis for targeted strategies.

Several limitations should be considered when interpreting these findings. The cross-sectional design precludes causal inference, and the direction of the relationships among job satisfaction, affective organizational commitment, career engagement, and quiet quitting cannot be established. Reciprocal or reverse pathways, for example quiet quitting gradually eroding satisfaction and attachment, are equally compatible with the data. Data were collected through convenience and snowball sampling in a single province, so the findings may not generalize to healthcare workers in other regions of Türkiye or in other countries. Because the survey link circulated through personal networks, the number of people who received an invitation is unknown, a response rate could not be calculated, and non-response bias cannot be assessed; the platform also did not technically prevent multiple submissions, so duplicate participation, although unlikely, cannot be fully ruled out. The sample was dominated by women and by nurses, while several occupational subgroups were very small (for example, only three laboratory technicians participated), which limits both the precision of profession-based comparisons and the generalizability of the results to the full range of healthcare occupations. Institution type was not recorded, so potential differences between public and private settings could not be examined. All constructs were measured with self-report instruments in a single survey, which raises the possibility of common method bias, although the statistical checks reported above suggest it was limited. In addition, although the two-dimensional structure of the quiet quitting scale was modeled and subdimension-level sensitivity analyses yielded the same pattern, the main analyses relied on the total score, whose work-related subdimension showed modest internal consistency and one weak item. Because the life-related subdimension broadens the operationalization beyond narrowly work-focused withdrawal, the findings pertain to quiet quitting as operationalized by this scale, and its differentiation from adjacent constructs such as burnout or general disengagement was not directly assessed. Moreover, the latent overlap between affective organizational commitment and quiet quitting was substantial, and at the subdimension level the HTMT value for commitment and work-related quiet quitting exceeded conventional thresholds, so their empirical distinctness should be read as relative rather than absolute. Accordingly, the mediation and moderated mediation estimates may partly reflect strongly overlapping measurement content—commitment and work-related quiet quitting behaving, in part, as opposite poles of an attachment–withdrawal continuum—rather than a fully distinct psychological mechanism. Measurement invariance of the scales across the compared demographic groups was not tested; establishing at least scalar invariance is recommended before interpreting group mean differences [39], so the demographic comparisons should be considered exploratory. Because measurement non-invariance can also inflate apparent between-group differences in structural parameters, recently proposed approaches such as stabilizer variables offer one route for separating genuine from measurement-induced heterogeneity in future multi-group applications [59]. Finally, parametric group comparison tests were applied although the score distributions departed from normality. This choice is supported by the sample size and by the use of bootstrap inference in the moderated mediation model, but the group comparison results should nevertheless be interpreted with caution.

## 5. Conclusions

The results show that job satisfaction, affective organizational commitment, and career engagement were consistently associated with lower quiet quitting among healthcare workers. Higher job satisfaction was associated with stronger affective organizational commitment, and stronger commitment was associated with less psychological withdrawal from work. Descriptively, nurses reported the lowest job satisfaction and the highest quiet quitting in our sample; although the difference in quiet quitting was not confirmed in pairwise comparisons, the pattern warrants further investigation.

The study’s central contribution is conditional. Rather than compensating for low affective organizational commitment, career engagement amplified the inverse association between commitment and quiet quitting, so that highly career-engaged employees showed the lowest quiet quitting when they felt attached to their organization and the highest when they did not. If this pattern is confirmed in longitudinal and intervention research, one implication would be that investments in employees’ career development may pay off for organizations mainly when they are paired with conditions that sustain emotional attachment, and that career support offered without such conditions may coincide with disengagement from the organization rather than protection against it.

These findings translate into cautious rather than prescriptive implications. Healthcare managers may consider combining efforts to improve working conditions and satisfaction with proactive support for employees’ career development, treating the two as complementary rather than alternative levers; our cross-sectional data cannot, however, establish that such programs would reduce quiet quitting, and possible downstream consequences for care quality and patient safety remain a broader concern raised in the literature rather than an outcome examined here. Future research should follow employees over time, measure career redirection and employability directly, record institution type, and test whether interventions that strengthen attachment and career development jointly reduce quiet quitting.

## Figures and Tables

**Table 1:** Participant Information.

Variable	Category	Frequency (f)	Percentage (%)
**Gender**	Woman	272	72.9
	Man	101	27.1
**Education**	High School	21	5.6
	University	240	64.3
	Master’s Degree	71	19
	Doctorate	10	2.7
	Medical Specialization	31	8.3
**Marital Status**	Married	212	56.8
	Single	161	43.2
**Profession**	Doctor	47	12.6
	Nurse	178	47.7
	Midwife	24	6.4
	Lab Technician	3	0.8
	Technician	12	3.2
	Associate technician	43	11.5
	Physiotherapist	18	4.8
	Other	48	12.9
**Income Level (TL)**	0–12,000	8	2.1
	12,001–24,000	52	13.9
	24,001–36,000	216	57.9
	36,001–48,000	47	12.6
	48,001 and above	50	13.4
**Total Professional Experience**	Less than 1 year	28	7.5
	1–5 years	111	29.8
	6–10 years	82	22
	11–15 years	45	12.1
	16–20 years	37	9.9
	21 years and above	70	18.8
**Total Professional Experience in the Institution**	Less than 1 year	74	19.8
	1–5 years	161	43.2
	6–10 years	58	15.5
	11–15 years	36	9.7
	16–20 years	22	5.9
	21 years and above	22	5.9
**Total**		373	100

**Table 2:** Fit Indices for the Hypothesized and Alternative Measurement Models.

Model	χ^2^_SB_ (df)	CFI	TLI	RMSEA [90% CI]	SRMR	AIC	BIC
Five-factor (hypothesized; QQ two subdimensions)	908.78 (340)	0.913	0.904	0.072 [0.067, 0.078]	0.063	27,151.35	27,410.17
Four-factor (QQ unidimensional)	1038.67 (344)	0.894	0.883	0.080 [0.074, 0.085]	0.070	27,300.28	27,543.42
Three-factor (AOC + QQ combined)	1196.85 (347)	0.869	0.857	0.088 [0.083, 0.093]	0.070	27,489.83	27,721.21
Three-factor (JS + AOC combined)	1256.78 (347)	0.859	0.847	0.091 [0.086, 0.097]	0.073	27,563.34	27,794.71
One-factor	2735.21 (350)	0.624	0.593	0.148 [0.143, 0.154]	0.121	29,369.89	29,589.50

n = 373. MLR estimation with robust fit indices; χ^2^_SB_ = Satorra–Bentler scaled chi-square. JS = job satisfaction; AOC = affective organizational commitment; QQ = quiet quitting. All scaled Δχ^2^ tests favored the five-factor model (*p* < 0.001).

**Table 3:** Descriptive Statistics, Reliability and Validity Coefficients, and Spearman Correlations Between the Study Variables.

Variable	M ± SD	Range	α	CR	AVE	1	2	3	4	4a
**1. Job satisfaction**	16.14 ± 5.20	5–25	0.91	0.91	0.66					
**2. Affective organizational commitment**	18.69 ± 6.28	6–30	0.90	0.90	0.60	0.722 **				
**3. Career engagement**	29.28 ± 8.81	9–45	0.94	0.94	0.63	0.497 **	0.442 **			
**4. Quiet quitting (total)**	27.37 ± 7.37	8–40	0.87	0.88	0.48	−0.703 **	−0.756 **	−0.454 **		
**4a. QQ towards work**	8.28 ± 3.05	3–15	0.64	0.69	0.46	−0.694 **	−0.696 **	−0.524 **	0.849 **	
**4b. QQ towards life**	19.09 ± 5.05	5–25	0.88	0.88	0.59	−0.582 **	−0.659 **	−0.353 **	0.910 **	0.577 **

n = 373. α = Cronbach’s alpha; CR = composite reliability; AVE = average variance extracted (total scores from the four-factor, subdimensions from the five-factor CFA solution). ** *p* < 0.001 (Spearman’s ρ).

**Table 4:** Comparison of Scale Scores by Demographic Characteristics.

Characteristic	Job Satisfaction	Affective Organizational Commitment	Career Engagement	Quiet Quitting
**Gender**				
Woman	15.70 ± 5.00	18.25 ± 6.09	29.06 ± 8.52	28.19 ± 6.97
Man	17.32 ± 5.56	19.88 ± 6.65	29.88 ± 9.57	25.14 ± 7.97
Test	t(371) = −2.69, *p* = 0.007 **, d = 0.31	t(371) = −2.25, *p* = 0.025 *, d = 0.26	t(371) = −0.80, *p* = 0.424, d = 0.09	t(160) = 3.40, *p* = 0.001 **, d = 0.42
**Marital status**				
Married	16.56 ± 5.07	19.07 ± 6.39	28.95 ± 8.75	27.25 ± 7.22
Single	15.58 ± 5.33	18.19 ± 6.12	29.72 ± 8.90	27.53 ± 7.58
Test	t(371) = 1.79, *p* = 0.074, d = 0.19	t(371) = 1.33, *p* = 0.184, d = 0.14	t(371) = −0.84, *p* = 0.403, d = 0.09	t(371) = −0.37, *p* = 0.714, d = 0.04
**Education**				
High school	17.38 ± 4.09	20.38 ± 5.02	28.10 ± 8.03	25.24 ± 6.24
University	15.81 ± 5.08	18.26 ± 6.12	28.69 ± 8.46	28.18 ± 6.45
Master’s degree	16.56 ± 5.00	19.14 ± 6.51	30.73 ± 9.51	27.30 ± 7.66
Doctorate	16.20 ± 6.36	18.00 ± 7.30	31.40 ± 11.09	24.30 ± 12.25
Medical specialty	16.84 ± 6.72	20.03 ± 7.30	30.65 ± 9.43	23.71 ± 10.39
Test	F(4, 368) = 0.80, *p* = 0.527, ω^2^ < 0.01	F(4, 368) = 1.14, *p* = 0.339, ω^2^ < 0.01	F(4, 368) = 1.18, *p* = 0.320, ω^2^ < 0.01	F(4, 368) = 3.60, *p* = 0.007 **, ω^2^ = 0.027
**Profession**				
Doctor	16.60 ± 6.16	19.17 ± 6.84	29.15 ± 9.11	25.40 ± 9.08
Nurse	14.77 ± 4.72	17.17 ± 5.92	28.02 ± 8.68	28.84 ± 6.70
Midwife	18.29 ± 5.40	21.04 ± 6.35	28.71 ± 9.63	26.17 ± 9.57
Laboratory technician	19.00 ± 3.61	22.00 ± 3.46	31.33 ± 12.10	20.67 ± 8.50
Technician	17.58 ± 6.57	19.25 ± 6.06	28.75 ± 10.16	26.67 ± 7.92
Associate technician	16.98 ± 4.87	19.91 ± 6.31	32.33 ± 8.98	27.14 ± 5.90
Physiotherapist	18.78 ± 5.89	20.22 ± 7.42	33.00 ± 9.52	23.67 ± 10.40
Other	17.40 ± 4.33	20.65 ± 5.61	30.27 ± 6.90	26.62 ± 5.05
**Test**	F(7, 365) = 4.11, *p* < 0.001 **, ω^2^ = 0.055	F(7, 365) = 3.32, *p* = 0.002 **, ω^2^ = 0.042	F(7, 365) = 1.88, *p* = 0.072, ω^2^ = 0.016	F(7, 365) = 2.76, *p* = 0.008 **, ω^2^ = 0.032
Monthly income (TL)				
0–12,000	15.38 ± 5.93	17.88 ± 6.56	31.75 ± 8.50	28.88 ± 6.85
12,001–24,000	17.50 ± 4.39	20.77 ± 4.69	30.73 ± 8.05	26.69 ± 5.41
24,001–36,000	15.45 ± 5.14	17.78 ± 6.36	28.40 ± 8.64	28.75 ± 6.47
36,001–48,000	16.28 ± 5.16	19.19 ± 6.26	30.06 ± 9.66	24.70 ± 8.80
48,001 and above	17.68 ± 5.72	20.10 ± 6.76	30.44 ± 9.37	24.38 ± 9.65
**Test**	F(4, 368) = 3.05, *p* = 0.017 *, ω^2^ = 0.022	F(4, 368) = 3.37, *p* = 0.010 *, ω^2^ = 0.025	F(4, 368) = 1.36, *p* = 0.248, ω^2^ < 0.01	F(4, 368) = 5.98, *p* < 0.001 **, ω^2^ = 0.051
Professional experience				
Less than 1 year	17.82 ± 4.55	20.71 ± 5.95	32.39 ± 8.40	24.71 ± 7.19
1–5 years	14.92 ± 5.52	16.61 ± 6.59	28.76 ± 9.16	29.12 ± 7.54
6–10 years	15.67 ± 5.14	18.37 ± 5.89	30.38 ± 7.85	27.16 ± 7.19
11–15 years	16.04 ± 5.60	18.40 ± 6.28	27.69 ± 8.49	28.18 ± 6.72
16–20 years	17.65 ± 4.12	20.81 ± 5.70	31.78 ± 8.00	25.08 ± 7.79
**21 years and above**	17.20 ± 4.81	20.61 ± 5.64	27.29 ± 9.51	26.59 ± 6.99
Test	F(5, 367) = 3.24, *p* = 0.007 **, ω^2^ = 0.029	F(5, 367) = 5.55, *p* < 0.001 **, ω^2^ = 0.057	F(5, 367) = 2.70, *p* = 0.021 *, ω^2^ = 0.022	F(5, 367) = 3.05, *p* = 0.010 *, ω^2^ = 0.027
**Institutional tenure**				
Less than 1 year	16.57 ± 5.45	19.09 ± 6.58	31.39 ± 9.13	26.01 ± 8.22
1–5 years	15.95 ± 5.36	18.39 ± 6.39	29.40 ± 8.98	27.46 ± 8.06
6–10 years	15.31 ± 5.47	17.97 ± 6.57	29.33 ± 8.29	28.62 ± 5.55
11–15 years	16.39 ± 4.47	17.92 ± 5.98	26.00 ± 8.28	27.89 ± 6.49
16–20 years	15.41 ± 4.24	19.73 ± 4.19	27.82 ± 6.79	27.32 ± 5.01
21 years and above	18.55 ± 3.91	21.68 ± 5.39	28.00 ± 9.27	27.14 ± 6.42
Test	F(5, 367) = 1.49, *p* = 0.192, ω^2^ < 0.01	F(5, 367) = 1.53, *p* = 0.180, ω^2^ < 0.01	F(5, 367) = 2.10, *p* = 0.065, ω^2^ = 0.015	F(5, 367) = 0.88, *p* = 0.495, ω^2^ < 0.01

Values are mean ± standard deviation. t = independent-samples t test (Welch correction for quiet quitting by gender), F = one-way ANOVA, d = Cohen’s d (absolute value), ω^2^ = omega squared. * *p* < 0.05, ** *p* < 0.01. All comparisons were exploratory. Mann–Whitney U, Welch ANOVA, and Kruskal–Wallis sensitivity tests confirmed the significant results, except quiet quitting by education (Welch *p* = 0.065, Kruskal–Wallis *p* = 0.184) and quiet quitting by profession (Welch *p* = 0.086). With Holm correction across all 28 omnibus tests, the differences that remained significant were affective organizational commitment by professional experience and by profession, job satisfaction by profession, and quiet quitting by income and by gender.

**Table 5:** PROCESS Macro Results.

Dependent Variables	Predictor Variables	B	SE	t	*p*	LLCI	ULCI
Affective Organizational Commitment (Mediator)	constant	4.4169	0.7236	6.1042	<0.001	2.9941	5.8398
	Job Satisfaction (X)	0.8844	0.0427	20.721	<0.001	0.8005	0.9684
		R^2^ = 0.536	F = 429.36		*p* < 0.001		
**Quiet Quitting (Dependent)**	constant	34.0720	1.1110	30.6682	<0.001	31.8873	36.2566
	Job Satisfaction (X)	−0.3814	0.0671	−5.6857	<0.001	−0.5134	−0.2495
	Affective Organizational Commitment (M)	−0.5139	0.0546	−9.4113	<0.001	−0.6213	−0.4066
	Career Engagement (W)	−0.1456	0.0304	−4.7900	<0.001	−0.2053	−0.0858
	**Interaction (M × W)**	**−0.0216**	**0.0039**	**−5.503**	**<0.001**	**−0.0293**	**−0.0139**
		R^2^ = 0.642	F = 165.35		*p* < 0.001		

B = unstandardized coefficient, SE = standard error, LLCI and ULCI = lower and upper limits of the 95% confidence interval. The mediator and moderator were mean-centered before forming the product term (M at 18.69, W at 29.28); the interaction coefficient and model fit are identical to the uncentered specification. All coefficients remained significant with HC3 heteroscedasticity-consistent standard errors.

## Data Availability

The data presented in this study are available from the corresponding author upon reasonable request. The data are not publicly available due to ethical restrictions imposed by the institutional ethics committee and the confidentiality of participant information.

## Supplementary Materials

The following supporting information can be downloaded at: https://www.mdpi.com/article/10.3390/healthcare14152366/s1, Table S1: STROBE Statement: Checklist of Items for Cross-Sectional Studies; Table S2: Games–Howell pairwise comparisons for significant omnibus tests (mean differences with 95% confidence intervals); Table S3: Standardized factor loadings and latent correlations from the confirmatory factor analysis (MLR estimation); Table S4: Heterotrait–monotrait (HTMT) ratios with percentile bootstrap 95% confidence intervals; Table S5: Additional psychometric analyses of the quiet-quitting scale (item statistics, McDonald’s omega, higher-order and bifactor indices, and measurement models excluding the weakly loading item); Table S6: Spearman correlations between the quiet-quitting items and the study variables; Figure S1: Residual diagnostics for the moderated mediation outcome model: normal Q–Q plot of residuals (left panel) and residuals versus fitted values (right panel); Figure S2: Simple slopes for the conditional effect of affective organizational commitment on quiet quitting at the 16th (W = 20), 50th (W = 29), and 84th (W = 38) percentiles of career engagement, with job satisfaction held at its mean; Figure S3: Normal Q–Q plots of the four scale totals.
