# Supplementary material for: Affective Organizational Commitment Links Job Satisfaction to Quiet Quitting in Healthcare Workers: The Amplifying Role of Career Engagement
Source: Healthcare (Basel). 2026 Aug 3;14(15):2366. doi: 10.3390/healthcare14152366 (PMC13464573; doi:10.3390/healthcare14152366)
Supplement: Supplementary file 1 [file healthcare-14-02366-s001.zip › healthcare-4449578-supplementary.pdf]

Supplementary Material for

Affective organizational commitment links job satisfaction to quiet quitting in healthcare workers: the amplifying role of career engagement

Sümeyra Gündem, Selma Söyük and Salim Yılmaz*

*: Corresponding author, salim.yilmaz@acibadem.edu.tr

Table S1. STROBE Statement: Checklist of Items for Cross-Sectional Studies

Item No	Section	Recommendation	Location in manuscript
1(a)	Title and abstract	Indicate the study's design with a commonly used term in the title or the abstract	Title; Abstract
1(b)		Provide in the abstract an informative and balanced summary of what was done and what was found	Abstract
2	Background/rationale	Explain the scientific background and rationale for the investigation being reported	Section 1
3	Objectives	State specific objectives, including any prespecified hypotheses	Sections 1.1-1.2 (H1-H4)
4	Study design	Present key elements of study design early in the paper	Section 2.1
5	Setting	Describe the setting, locations, and relevant dates, including periods of recruitment and data collection	Sections 2.2-2.3
6(a)	Participants	Give the eligibility criteria, and the sources and methods of selection of participants	Section 2.2
7	Variables	Clearly define all outcomes, exposures, predictors, potential confounders, and effect modifiers; give diagnostic criteria, if applicable	Sections 1.1, 2.4
8	Data sources/ measurement	For each variable of interest, give sources of data and details of methods of assessment (measurement)	Sections 2.3-2.4
9	Bias	Describe any efforts to address potential sources of bias	Section 2.5 (common method bias, diagnostics); Section 4 (limitations)
10	Study size	Explain how the study size was arrived at	Section 2.2
11	Quantitative variables	Explain how quantitative variables were handled in the analyses; describe which groupings were chosen and why	Sections 2.4-2.5
12(a)	Statistical methods	Describe all statistical methods, including those used to control for confounding	Section 2.5
12(b)		Describe any methods used to examine subgroups and interactions	Section 2.5
12(c)		Explain how missing data were addressed	Section 2.2
12(d)		If applicable, describe analytical methods taking account of sampling strategy	Section 2.2 (convenience and snowball sampling; no weighting applied)
12(e)		Describe any sensitivity analyses	Section 2.5
13	Participants	Report numbers of individuals at each stage of the study and reasons for non-participation	Section 2.2
14	Descriptive data	Give characteristics of study participants and information on exposures and potential confounders	Section 3, Tables 1 and 3
15	Outcome data	Report numbers of outcome events or summary measures	Section 3, Table 3
16	Main results	Give unadjusted and, if applicable, confounder-adjusted estimates and their precision (e.g., 95% confidence intervals)	Section 3, Tables 2-5, Figure 2
17	Other analyses	Report other analyses done, e.g., analyses of subgroups and interactions, and sensitivity analyses	Section 3 (robustness checks); Supplementary Materials
18	Key results	Summarise key results with reference to study objectives	Section 4
19	Limitations	Discuss limitations of the study, taking into account sources of potential bias or imprecision	Section 4 (Limitations)
20	Interpretation	Give a cautious overall interpretation of results considering objectives, limitations, multiplicity of analyses, and other relevant evidence	Section 4
21	Generalisability	Discuss the generalisability (external validity) of the study results	Section 4 (Limitations)
22	Funding	Give the source of funding and the role of the funders for the present study	Funding statement

Table S2. Games-Howell pairwise comparisons for significant omnibus tests (mean differences with 95% confidence intervals)

Variable	Comparison	Difference	95% CI	p (adj.)
Job satisfaction	Profession: Nurse vs. Other	2.63	0.39, 4.86	.010
Affective organizational commitment	Profession: Nurse vs. Other	3.47	0.59, 6.35	.008
Job satisfaction	Income: group 2 vs. 3	-2.05	-4.01, -0.09	.035
Affective organizational commitment	Income: group 2 vs. 3	-2.99	-5.16, -0.82	.002
Quiet quitting	Income: group 3 vs. 4	-4.04	-7.87, -0.22	.033
Quiet quitting	Income: group 3 vs. 5	-4.37	-8.40, -0.33	.028
Job satisfaction	Experience: 1-5 vs. 16-20 years	2.73	0.23, 5.23	.024
Job satisfaction	Experience: 1-5 vs. 21 years and above	2.28	0.04, 4.52	.044
Affective organizational commitment	Experience: <1 vs. 1-5 years	-4.10	-7.93, -0.27	.029
Affective organizational commitment	Experience: 1-5 vs. 16-20 years	4.20	0.90, 7.50	.005
Affective organizational commitment	Experience: 1-5 vs. 21 years and above	4.00	1.35, 6.65	<.001

Differences are the mean of the second listed group minus the first; income group numbers correspond to the categories in Table 1 of the manuscript.

Table S3. Standardized factor loadings and latent correlations from the confirmatory factor analysis (MLR estimation).

**Panel A. Five-factor solution — loadings**				**Panel B. Four-factor solution (composite scores)**			
**Construct**	**Item**	**Loading**	**SE**	**Construct**	**Item**	**Loading**	**SE**
Job satisfaction	it1	.861	.018	Job satisfaction	it1	.855	.019
	it2	.902	.015		it2	.908	.014
	it3	.689	.042		it3	.691	.041
	it4	.817	.023		it4	.816	.024
	it5	.786	.032		it5	.785	.032
Affective organizational commitment	ob1	.734	.030	Affective organizational commitment	ob1	.733	.030
	ob2	.676	.035		ob2	.678	.035
	ob3	.768	.042		ob3	.768	.042
	ob4	.863	.021		ob4	.863	.022
	ob5	.822	.027		ob5	.816	.027
	ob6	.774	.038		ob6	.780	.038
Career engagement	ka1	.712	.034	Career engagement	ka1	.712	.034
	ka2	.890	.015		ka2	.890	.015
	ka3	.911	.016		ka3	.912	.016
	ka4	.884	.018		ka4	.884	.018
	ka5	.648	.039		ka5	.647	.039
	ka6	.778	.028		ka6	.777	.028
	ka7	.784	.027		ka7	.784	.027
	ka8	.767	.029		ka8	.767	.029
	ka9	.752	.033		ka9	.752	.033
Quiet quitting – work-related	si1	.331	.059	Quiet quitting	si1	.330	.052
	si2	.785	.032		si2	.739	.030
	si3	.803	.029		si3	.706	.031
Quiet quitting – life-related	si4	.814	.029		si4	.794	.029
	si5	.834	.028		si5	.798	.032
	si6	.677	.043		si6	.623	.050
	si7	.797	.027		si7	.796	.027
	si8	.695	.043		si8	.646	.048

**Latent correlations**	**1**	**2**	**3**	**4**	**5**	**Latent correlations**	**1**	**2**	**3**	**4**
1. Job satisfaction						1. Job satisfaction				
2. Affective organizational commitment	.796					2. Affective organizational commitment	.796			
3. Career engagement	.501	.468				3. Career engagement	.501	.468		
4. Quiet quitting – work-related	−.858	−.882	−.562			4. Quiet quitting	-.759	-.812	-.517	–
5. Quiet quitting – life-related	−.671	−.730	−.455	.785	–	–	–	–	–	–

All loadings are significant at $p < .001$. Panel A reports the preferred five-factor solution, in which quiet quitting is represented by its two subdimensions; Panel B reports the four-factor solution corresponding to the four composite scores used in the main analyses. In Panel B, the quiet-quitting items load on a single factor corresponding to the total score used in the main analyses.

Table S4. Heterotrait–monotrait (HTMT) ratios of correlations with percentile bootstrap 95% confidence intervals

Construct pair	HTMT	95% CI	Level
JS – AOC	.810	[.745, .868]	Composite
JS – CE	.537	[.445, .623]	Composite
JS – QQ (total)	.766	[.713, .817]	Composite
AOC – CE	.504	[.408, .591]	Composite
AOC – QQ (total)	.822	[.775, .865]	Composite
CE – QQ (total)	.569	[.475, .651]	Composite
JS – QQ-work	.916	[.835, 1.001]	Subdimension
JS – QQ-life	.648	[.585, .708]	Subdimension
AOC – QQ-work	.930	[.858, 1.004]	Subdimension
AOC – QQ-life	.720	[.663, .770]	Subdimension
CE – QQ-work	.714	[.614, .808]	Subdimension
CE – QQ-life	.466	[.357, .560]	Subdimension
QQ-work – QQ-life	.839	[.759, .922]	Subdimension
AOC – QQ (7 items, item 1 excluded)	.817	[.771, .860]	Item 1 excluded
AOC – QQ-work (2 items, item 1 excluded)	.879	[.815, .942]	Item 1 excluded

n = 373. HTMT values were computed from the observed item correlations; 95% confidence intervals are percentile bootstrap intervals (5,000 resamples). JS = job satisfaction; AOC = affective organizational commitment; CE = career engagement; QQ = quiet quitting. At the composite level, HTMT2 (geometric-mean) values were identical to the reported values at three decimals. Values below .85 satisfy the strict criterion and values below .90 the lenient criterion; the AOC–QQ-work and JS–QQ-work ratios exceed both thresholds and are discussed in the manuscript. At the composite level, the upper confidence limits for the JS–AOC and AOC–QQ (total) pairs slightly exceed .85 while remaining below .90.

Table S5. Additional psychometric analyses of the quiet-quitting scale

Panel A. Work-related subdimension (3 items) — item statistics

Item	Mean (SD)	Corrected item–total r	Alpha if item deleted
Item 1	2.51 (1.28)	.25	.78
Item 2	2.97 (1.33)	.57	.37
Item 3	2.80 (1.39)	.56	.37

Statistic	Estimate
Mean inter-item correlation (work)	.37
Cronbach's α (work, 3 items)	.64
McDonald's ω (work, 3 items)	.69
Item 2–Item 3 correlation	.64
Spearman–Brown reliability (Items 2–3)	.78
Cronbach's α (life, 5 items)	.88
McDonald's ω (life, 5 items)	.88

Panel B. Higher-order and bifactor representations of quiet quitting

Statistic	Estimate
$\Delta\chi^2(2)$ vs. correlated five-factor model	1.33 (p = .514)
ΔCFI	< .001
Higher-order loading, work	.99
Higher-order loading, life	.80

Fit and Reliability Indices	Higher-order model (second-order QQ factor)	Bifactor model (8 items)
χ^2SB(df)	910.47(342)	21.67(12)
CFI	.913	.991
TLI	.904	.979
RMSEA [90% CI]	.072 [.066, .078]	.053 [.010, .088]
SRMR	.063	.029
ωt	—	.90
ωH	—	.76
ECV	—	.72
ωHS, work-specific factor	—	.15
ωHS, life-specific factor	—	.24

Panel C. Measurement models excluding item 1

Fit index	Five-factor model	Four-factor model
χ^2SB (df)	850.21 (314)	982.39 (318)
CFI	.917	.897
TLI	.907	.886
RMSEA	.073	.081
90% CI for RMSEA	[.067, .079]	[.075, .087]
SRMR	.060	.068
Reliability and validity with item 1 removed		
Cronbach's α, QQ total (7 items)	.89	
CR, QQ total (7 items)	.89	
AVE, QQ total (7 items)	.54	
CR, QQ-work (2 items)	.78	
AVE, QQ-work (2 items)	.64	
Loadings, QQ-work	.79, .82	
Latent r, AOC – QQ-work	−.88	

Panel D. Factor-score sensitivity analysis of the moderated mediation model

Parameter	Estimate
Interaction, AOC × CE (B)	−0.137 (SE = 0.024, p < .001)
Indirect effect, 16th percentile of CE	−0.297
Indirect effect, 50th percentile of CE	−0.416
Indirect effect, 84th percentile of CE	−0.530
Index of moderated mediation	−0.116 [−0.155, −0.077]

n = 373. M = mean; SD = standard deviation. MLR estimation unless stated otherwise; CR = composite reliability; AVE = average variance extracted; ωH = hierarchical omega; ωHS = hierarchical omega of the specific factor; ECV = explained common variance; AOC = affective organizational commitment; CE = career engagement; QQ = quiet quitting. The bifactor model specified a general factor plus orthogonal work- and life-specific factors. Confidence intervals in Panel D are percentile bootstrap intervals (5,000 resamples). The higher-order model fit the data as well as the correlated five-factor model, and in the bifactor solution reliable variance in the total score was carried predominantly by the general factor; re-estimation with factor scores reproduced the pattern of the composite-score analysis.

Table S6. Spearman correlations between the quiet-quitting items and the study variables

Item	Item wording (English translation)	JS	AOC	CE
Item 1 (work)	If I quit my job, it would be very difficult for me to find a new one	−.29	−.28	−.37
Item 2 (work)	I don't see a future for my career at this workplace	−.62	−.64	−.49
Item 3 (work)	I feel undervalued at the company where I work	−.69	−.69	−.40
Item 4 (life)	Life isn't just about work; I don't agree with the company's policy of long working hours	−.58	−.57	−.36
Item 5 (life)	I hate it when meetings are scheduled right before the end of the workday and when my manager calls me outside of work hours	−.53	−.57	−.30
Item 6 (life)	My health and well-being come before my career	−.29	−.36	−.25
Item 7 (life)	No matter how hard I work, my managers' expectations of me never end	−.56	−.59	−.37
Item 8 (life)	I believe that the workplace of the future should be designed with the employee in mind, not the employer	−.31	−.42	−.21
Mean |ρ|, work items	—	.53	.54	.42
Mean |ρ|, life items	—	.45	.50	.30

n = 373. All correlations p < .001. JS = job satisfaction; AOC = affective organizational commitment; CE = career engagement. English translations of the administered items are shown; the scale was developed and administered in Turkish [38]. Life-related items correlated substantially with the work attitudes, indicating that the life-related content of the scale remains anchored in work-related withdrawal.

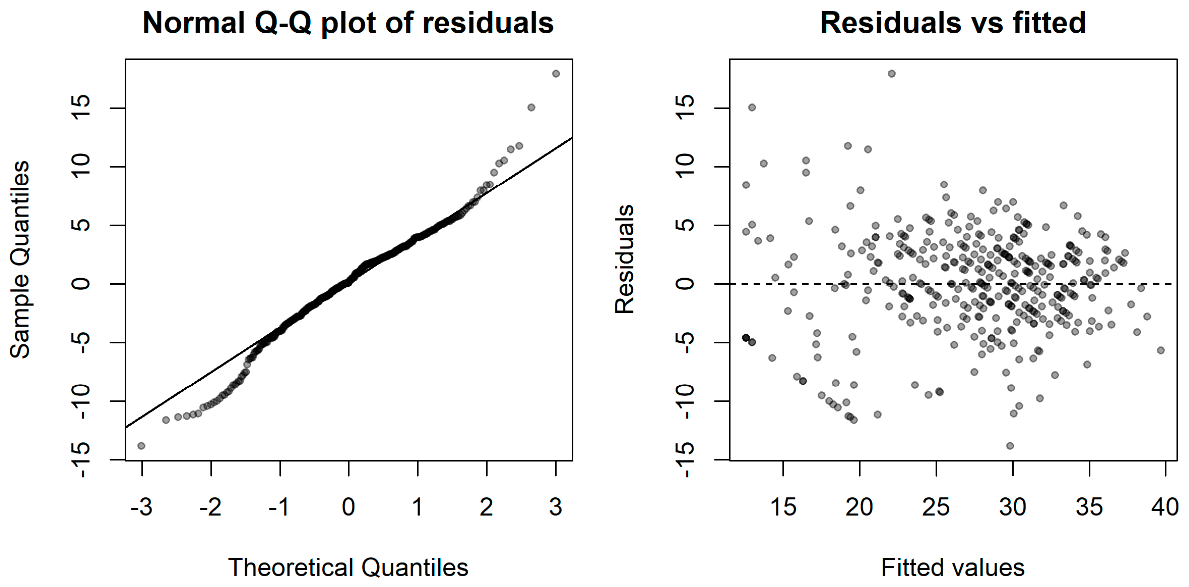

Figure S1. Residual diagnostics for the moderated mediation outcome model: normal Q-Q plot of residuals (left panel) and residuals versus fitted values (right panel).

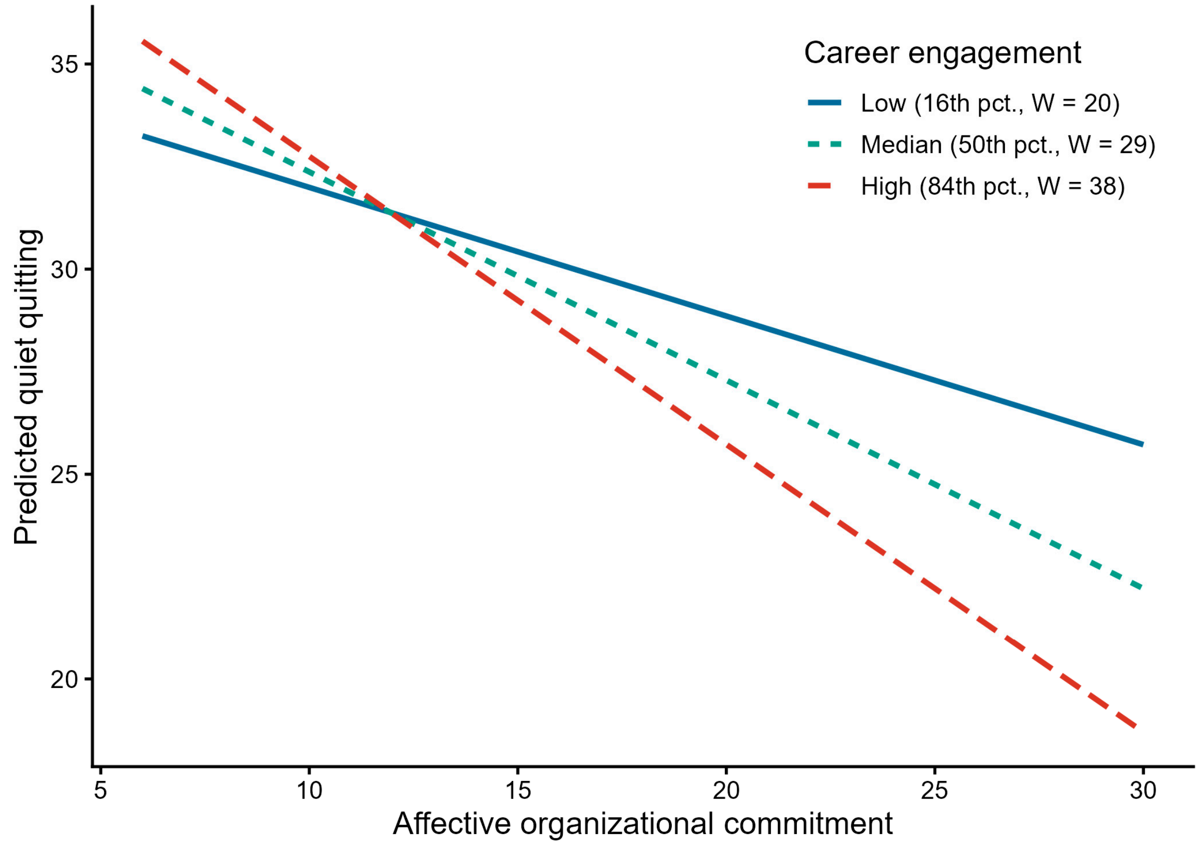

Figure S2. Simple slopes for the conditional effect of affective organizational commitment on quiet quitting at the 16th (W = 20), 50th (W = 29), and 84th (W = 38) percentiles of career engagement, with job satisfaction held at its mean.

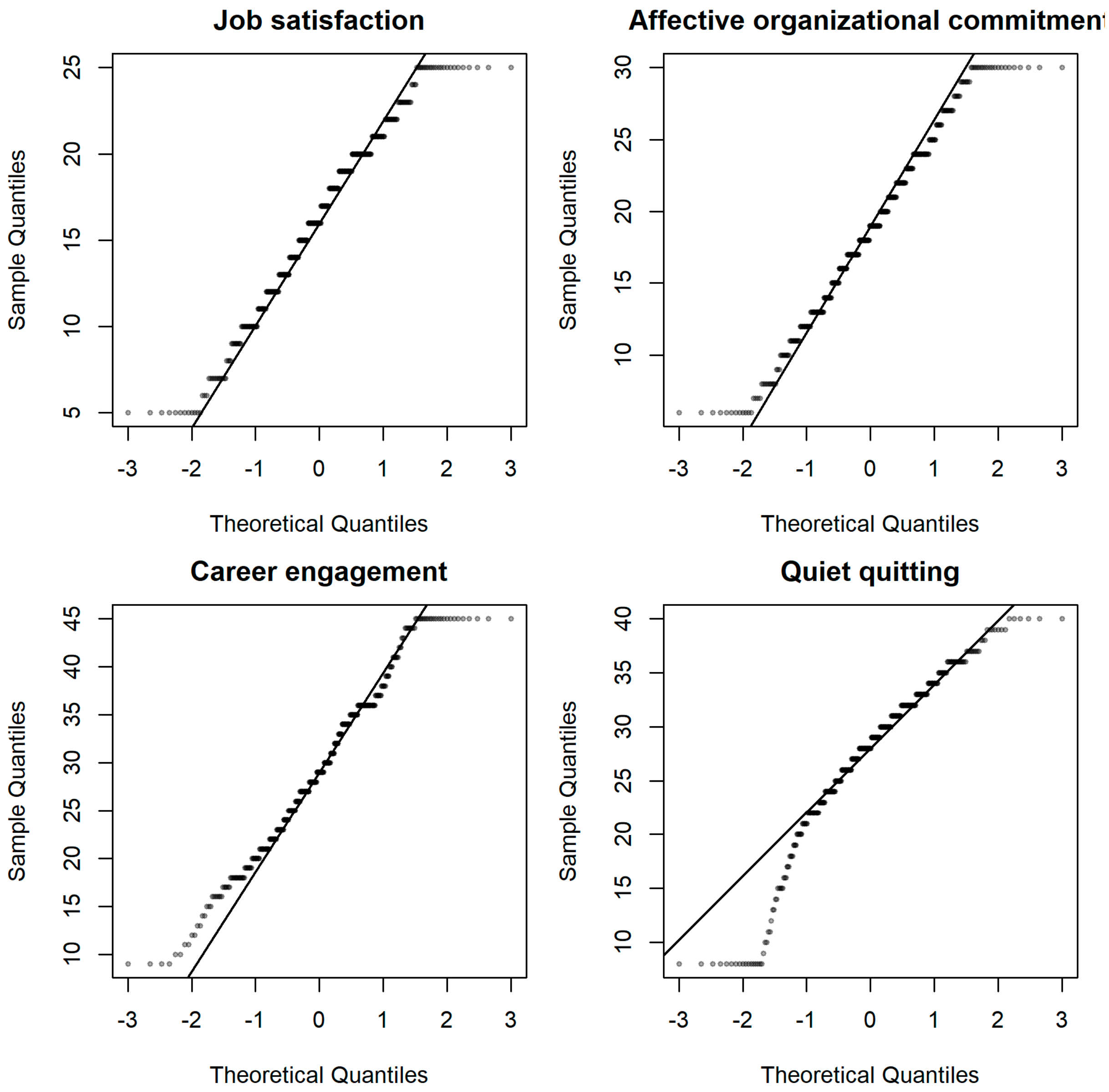

Figure S3. Normal Q-Q plots of the four scale totals.
